# *Hirsutella Sinensis* Fungus Regulates CD8^+^ T Cell Exhaustion Through Involvement of T-Bet/Eomes in the Tumor Microenvironment

**DOI:** 10.3389/fphar.2020.612620

**Published:** 2021-01-08

**Authors:** Lu Jin, Lushuai Jin, Renjie Wu, Xia Liu, Xinhai Zhu, Qiyang Shou, Huiying Fu

**Affiliations:** ^1^ The Second Clinical Medical School, Zhejiang Chinese Medical University, Hangzhou, China; ^2^ School of Pharmacy, Zhejiang Chinese Medical University, Hangzhou, China; ^3^ Department of Medicine, Zhejiang Academy of Traditional Chinese Medicine, Hangzhou, China; ^4^ Department of Thoracic Surgery, Zhejiang Hospital, Hangzhou, China

**Keywords:** Cordyceps sinensis, *in vivo* imaging technology, exhausted T cells, Inhibitory receptors, T-bet/Eomes

## Abstract

**Background:** Targeting exhausted T (Tex) cells is a promising strategy for anti-tumour treatment. Previously, we demonstrated that *Hirsutella sinensi*s fungus (HSF) could significantly increase T cell infiltration and the effector T cell ratio in the tumor microenvironment, activating systemic immune responses. However, we do not know how HSF regulates Tex cells in the tumor microenvironment. Here, we explored the mechanism underlying HSF inhibition of Tex cells and tumor growth and metastasis in breast cancer.

**Methods:** We examined the effects of HSF on various tumor mouse models using *in vivo* imaging technology. Lung metastasis was detected by H&E staining and the T cell subsets in the tumor microenvironment were assayed with flow cytometry. The *in vitro* proliferation, function and apoptosis of CD8^+^ T cells were measured, as well as the *T-bet* and *PD-1* mRNA expressions.

**Results:** HSF inhibited tumor growth and lung metastasis in the mice, and had significantly higher CD44^Low^CD62L^Hi^ and CD44^Hi^CD62L^Low^populations in the tumour-infiltrating CD8^+^ T cells. However, HSF significantly reduced levels of inhibitory receptors, such as PD-1, TIGIT, CTLA-4, and regulatory T cells. *In vitro*, HSF inhibited the CD8^+^ T cell apoptosis rate, and promoted CD8^+^ T cell proliferation and secretion of interferon (IFN)-γ and granzyme B. Furthermore, HSF treatment both *in vivo* and *in vitro* significantly increased Eomes expression, while decreasing T-bet expression.

**Conclusion:** HSF exerted anti-tumour effects mainly through the immune system, by promoting effector/memory T cells and reducing Tex cell production in the tumor microenvironment. The specific mechanisms involved inhibiting T-bet and promoting Eomes to decrease the expression of immune inhibitor receptors and enhance the T cell function, respectively.

## Introduction

During acute infection or vaccinations, partly functional effector CD8^+^ T cells are naturally transformed into memory CD8^+^ T cells after viral clearance. In contrast, during chronic infections or cancers, the presence of persistent antigens hampers the proper development of CD8^+^ memory T cells, generating exhausted T (Tex) cells instead (Wherry, 2011; Jiang et al., 2015; Mclane et al., 2019; ; Philip and Schietinger, 2019). Tex cells have a unique differentiation, phenotype, and function, along with stable epigenetic inheritance. The action of the transcription factor Tox partially locks Tex cells during the early stages of infection (Mann and Kaech, 2019). Specific deletion of Tox in tumour-specific T cells eliminates the T cell exhaustion program, as inhibitory receptors (e.g. Pdcd1, Cd244, and Tigit) (Khan et al., 2019; Alfei et al., 2019) can no longer be upregulated. However, tumour-specific T cells lacking Tox fail to persist in the tumor. Therefore, in conditions such as cancer with chronic antigen stimulation, T cell exhaustion appears to be a self-protection measure that prevents excessive stimulation and activation-induced T cell death (Mclane et al., 2019). However, Tex cells restrict pathogen infections and immune responses to tumors, thereby limiting immune-mediated pathological damage to cancers (Mann and Kaech, 2019). This response often leads to continuous disease progression, meaning that Tex cells play a central role in the development of cancer and chronic infections (Khan et al., 2019). Fortunately, this exhaustion can be reversed, at least in part, mainly by blocking inhibitory pathways such as PD-1 (Wherry, 2011). Thus, how to better and reverse the Tex cells is currently a key issue in tumor immunotherapy.

*Hirsutella sinensis* fungus (HSF) is an artificial fermentation product of *Cordyceps sinensis* and is widely used in China as a substitute for the latter Li et al., 2020 and Zhang et al., 2020. The fermentation product can improve immunity and enhance disease resistance in postoperative or chemoradiotherapy patients (Liu et al., 2017; Yan et al., 2020). Our previous studies proved that HSFs can significantly increase T cell infiltration and effector T cell ratio in tumors and improve the immunosuppressive tumor environment that is considered to be the key factor of immunotherapy tolerance (Fu et al., 2018; Yan et al., 2020). Additionally, HSF can activate systemic immune responses, suggesting it as a useful drug in combination with immunotherapy, as effective cancer immunotherapy requires systemic immunity (Spitzer et al., 2017; Allen et al., 2020). However, we currently have little insight regarding how HSF regulates Tex cells in the tumor microenvironment and its potential active mechanism.

In this study, we investigated whether HSF inhibited tumor growth and metastasis through the immune system using imaging technology *in vivo*. We also analyzed the T cell subsets and the relationship to HSF mechanisms in the tumor microenvironment and *in vitro*. Our results provide insights into the applications of HSF in tumour-immune treatment.

## Methods

### Reagents and Chemicals

HSF was provided by Hangzhou KSBIO Science and Technology (BinJiang, Zhejiang, China). Quality control was performed following published methods (Fu et al., 2011).

The anti-mouse antibodies for fluorescence-activated cell sorting (FACS) were as follows: anti-CD45 FITC, anti-CD8 APC-H7, anti-Eomes PE-Cy7, anti-T-bet BV421, anti-PD-1 BV605, anti-CD4 PE, anti-CD44 BV510, and anti-CD62L PE-Cy7 from BD Biosciences.

### Cell Culture and CD8^+^ T Cells Isolation

The 4T1-Luc cells were cultured in IMDM medium supplemented with 10% fetal bovine serum, 50 IU/ml penicillin, and 50 μg/ml streptomycin (Hyclone, Logan, UT, USA) in a 5% CO_2_ incubator at 37 °C.

CD8^+^ T cells were isolated from splenocytes in C57/BL mice. Cells were magnetically labeled with anti-CD8a beads and isolated from spleen cell suspension using an LS Column (Miltenyi Biotec) placed in the magnetic field of a MACS separator. The process retained magnetically labeled CD8^+^ T cells in the column, and was then rinsed vigorously using 5 ml MACS buffer. CD8^+^ T cells were used for CFSE proliferation assays.

### Animal Experiments

We investigated HSF anti-breast cancer activity using spontaneous, postoperative metastasis and subcutaneous inoculation models. The protocol was approved by the Committee on the Ethics of Animal Experiments of Zhejiang Chinese Medical University (Number of resolution: ZSLL-2017–178). Spontaneous PyMT mice were obtained from Jackson Labs and bred at the Animal Center of Zhejiang University of Traditional Chinese Medicine. Twenty 6–8-week-old female PyMT mice (18–20 g) were randomly divided into two groups: model (distilled water) and HSF (6 g/kg, refer to our previously published article by Fu et al.). Animals were given HSF and water daily for 44 days. On days 0, 7, 15, 22, 29, 37, 44, and 47, tumor measurements were taken with Vernier calipers. Tumor volume was calculated as V = (length × width^2^)/2. Mice were sacrificed with CO_2_ asphyxiation on day 47 for lung collection.

To generate a postoperative metastasis breast cancer model, 20 female BALB/C mice (6–8-week-old, 18–20 g) were obtained from the Shanghai Laboratory Animal Center (Shanghai, China). They were housed under standard conditions. Their bottom-right second mammary fat pad was orthotopically injected with 5 × 10^5^ 4T1-luc cells in 100 µL PBS. After 3 weeks, tumors grew to an average volume of 250 mm. The mice were divided into model and HSF groups (n = 10 per group) based on their tumor volume. Tumors *in situ* were then surgically removed. On the following day, distilled water and HSF (6 g/kg) were intragastrically administered to the model and HSF groups, respectively, for four weeks.

To generate the subcutaneous inoculation model, 20 6–8-week-old female nude mice (18–20 g in weight) were injected with 4T1-luc cells (5 × 10^5^) in 100 µL PBS into the left armpit. On the following day, mice were randomly divided into model (distilled water) and HSF (6 g/kg HSF) groups. The mice were imaged on days 0, 4, 7, 11, and 14 for each group. Before imaging, mice were intraperitoneally injected with fluorescein substrate (150 mg/kg). At 8 min after the injection, the mice were anesthetized with isoflurane using an anesthesia machine (Summit Anesthesia, Salt Lake, UT, USA). They were then placed on a Xenogen IVIS 200 imaging system (Caliper Life Sciences, Hopkinton, MA, USA) to acquire *in vivo* images for measuring the primary-breast-cancer tumor size. Data analysis was performed using LT Living Image 4.3.

### Apoptosis Assay *in vitro*

Apoptosis experiments were performed using the Annexin V-FITC Apoptosis Detection Kit (BD Biosciences). Briefly, activated CD8^+^ T cells were cultured *in vitro* for one or 3 days. Cells were washed twice with pre-cooled PBS, then centrifuged at 300 g for 5 min, and 300 μL 1x binding buffer was added to the suspended cells. Cells were stained with 5 µL Annexin V-FITC for 15 min (in darkness, at 25 °C). Next, 5 μL PI stain was added, followed by washing twice with PBS. Before apoptosis detection, 200 μL 1x binding buffer was added to resuspend cells.

### CFSE Proliferation Assay *in vitro*

CD8^+^ T cells were purified and resuspended in PBS, then stained with carboxyfluorescein succinimidyl ester (CFSE; ThermoFisher Scientific) for 10 min at 37 °C. The reaction was stopped with CTL medium (RPMI 1640, 10% heat-inactivated fetal calf serum, 1% penicillin/streptomycin, 1% l-glutamine, 1% nonessential amino acids, 1% sodium pyruvate, and 0.1% β-mercaptoethanol), then resuspended in CTL medium. Cells were seeded (1 × 10^5^ cells/well) in 96-well plates coated with CD3 antibodies, and then HSF (0.2 mg/ml) was added to the medium. After culturing for 3 days, cells were collected for detection by flow cytometry.

### Functional Assay of CD8^+^ T Cell *in vitro*

CD8^+^ T cells (1 × 10^5^/well) were seeded in 96-well plates coated with CD3/CD28 antibodies and cultured for 3 days. Cells were then treated with PMA (40 ng/ml), ionomycin (2 μmol/L), and monensin (1.4 μL/ml) for 4 h at 37 °C with 5% CO_2_. They were then collected and fixed with Cytofix/Cytoperm (BD Bioscience) for 40 min at 4 °C. After washing twice, cells were stained with granzyme B and IFN-γ for 30 min at 4 °C, and resuspend in FACS buffer to assay.

### Flow Cytometry Assay

Tumor or spleen samples were placed into a six-well plate containing 2 ml of Harvest medium (RPMI 1640, 2% heat-inactivated fetal calf serum, 1% penicillin/streptomycin), and then ground into a single cell suspension using a grinding rod. After filtering with a 70 mM strainer, cells were placed on ice in FACS buffer (PBS, 2% heat-inactivated fetal calf serum, 1% penicillin/streptomycin, and 0.1% sodium azide) for 30 min of staining with surface antibodies. For intranuclear staining, cells were fixed with Cytofix/Cytoperm (BD Bioscience) for 40 min at 4 °C and then stained with intracellular antibodies (T-bet, Eomes). After washing twice with FACS buffer, samples were detected with FACS Canto II Cytometry (BD, USA), and the data were analyzed in FlowJo.

### Hematoxylin-Eosin Staining of Lung Tissues

Lung tissue was dissected and embedded in paraffin, cut into 4 μm sections, and stained with hematoxylin and eosin (H&E). Sections were imaged using a NanoZoomer Digital Slice Scanner (NDP; Nikon, Tokyo, Japan) and analyzed in NDP. view.

### RNA Purification and Quantitative PCR

CD8^+^ cells were treated with HSF (0.2 mg/ml) for 3 days before detection by qPCR. Total RNA was extracted using TRIzol (Invitrogen), then reverse-transcribed to cDNA using the PrimeScript RT Reagent Kit (Takara, USA). Samples were amplified using the TB Green PCR Master Mix kit (Takara) on an ABI 7900HT Fast Real-Time PCR System (Applied Biosystems, USA). Relative mRNA expression was normalized to β-actin and calculated using the 2^−ΔΔCt^ method. The primer sequences were as follows: T-bet Forward: GAT​CAC​TCA​GCT​GAA​AAT​CGA​C, T-bet Reverse: AGG​CTG​TGA​GAT​CAT​ATC​CTT​G; PD-1 Forward: ATG​ACT​TCC​ACA​TGA​ACA​TCC​T, PD-1 Reverse: CTC​CAG​GAT​TCT​CTC​TGT​TAC​C; β-actin Forward: GTG​ACG​TTG​ACA​TCC​GTA​AAG​A; β-actin Reverse: GCC​GGA​CTC​ATC​GTA​CTC​C.

### Statistical Analysis

Data are shown as means ± SEM. Between-group differences were determined using unpaired t-tests in SPSS 18.0. Significance was set at *p* < 0.05.

## Results

### HSF Inhibited Tumor Growth and Lung Metastasis in Mice Through the Immune System

**FIGURE 1:**
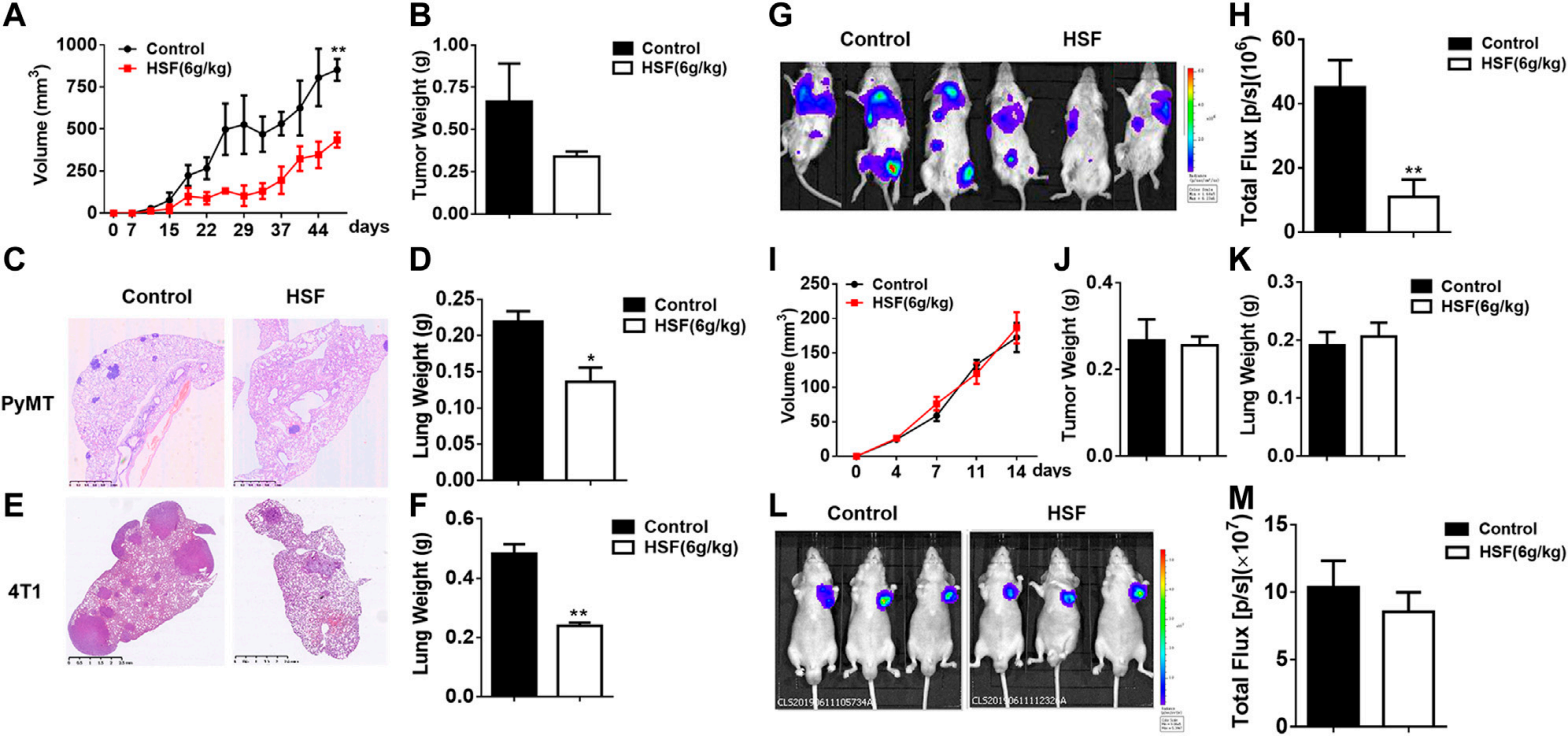
Anti-tumour effects of HSF in different mouse models of breast cancer. **(A)** Tumor volumes in PyMT mice over time. **(B)** Tumor weights in PyMT mice. **(C)** Representative image of pathological H&E staining in the lung tissue of control and HSF-treated PyMT mice. **(D)** Lung weights in PyMT mice. **(E)** Representative image of pathological H&E staining in the lung tissue from the postoperative metastasis mice. **(F)** Lung weights of the postoperative metastasis mice. **(G)** Representative luciferase image of 4T1-Luc tumor cell lung metastasis. **(H)** Quantitative statistics of lung metastasis luciferase imaging. **(I)** Tumor volumes in nude mice over time. **(J)** Tumor weights in nude mice. **(K)** Lung weights in nude mice. **(L)** Representative luciferase image of 4T1-Luc subcutaneous tumor. **(M)** Quantitative statistics of subcutaneous tumor luciferase imaging. Statistical analysis was performed with unpaired t-tests. Values are means ± SEM; n ≥ 6 mice/group; *p < 0.05, **p < 0.01, ***p < 0.001.

As shown in Figure 1, in a breast cancer model of mouse mammary tumor virus-polyomavirus middle T-antigen (MMTV-PyMT), tumor volume were significantly lower in the HSF-treated group than in the control group (Figures 1A,B), and tumors had more fluid-like substances in the control group, tumor weight were lower in the HSF-treated group. In addition, lung metastasis was significantly alleviated in the HSF-treated PyMT mice (Figure 1C) and postoperative metastasis mice (Figure 1E). These results were further confirmed with lung weight (Figures 1D,F). These results were consistent with the luciferase assay (Figures 1G,H). However, HSF treatment did not alter tumor volume, tumor weight, or lung weight in nude mice (Figures 1I–K), which was further confirmed by *in vivo* imaging technology (Figures 1L,M).

### HSF Increased Effector/Memory T Cells and Decreased Regulatory T Cells in the Tumor Microenvironment

**FIGURE 2:**
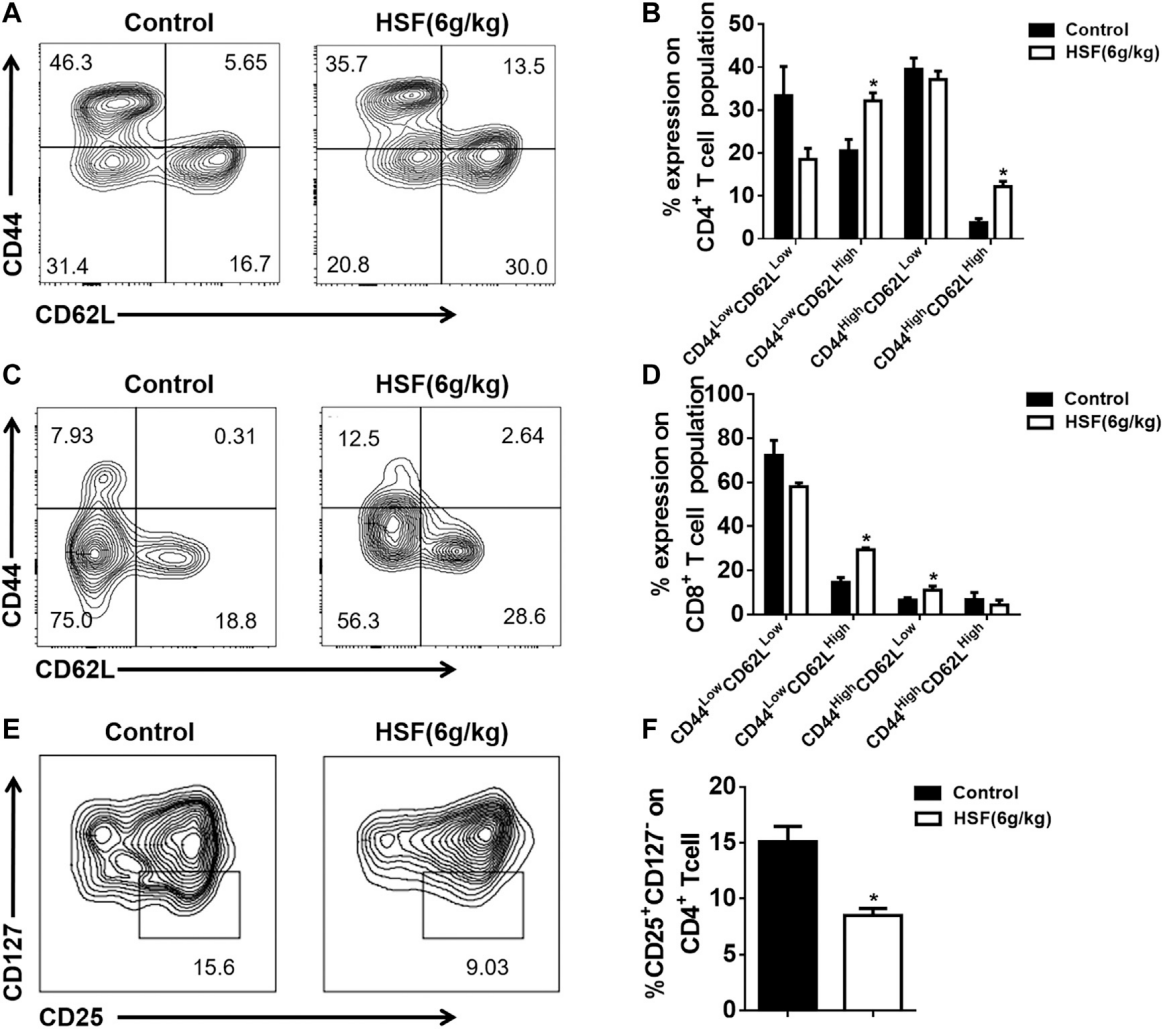
Effect of HSF on CD4^+^, CD8^+^, and regulatory T cells in the lung tumor microenvironment of postoperative metastasis mice. **(A)** Representative image of CD4^+^ CD44^Hi^CD62L^Low^, CD4^+^ CD44^Hi^CD62L^Hi^, CD4^+^ CD44^Low^CD62L^Hi^, CD4^+^ CD44^Low^CD62L^Low^ population. **(B)** Flow cytometry results of CD4^+^ CD44^Hi^CD62L^Low^, CD4^+^ CD44^Hi^CD62L^Hi^, CD4^+^ CD44^Low^CD62L^Hi^, CD4^+^ CD44^Low^CD62L^Low^, expressed as percentage of tumour-infiltrating CD4^+^ T cells. **(C)** Representative image of CD8^+^ CD44^Hi^CD62L^Low^, CD8^+^ CD44^Hi^CD62L^Hi^, CD8^+^ CD44^Low^CD62L^Hi^, CD8^+^ CD44^Low^CD62L^Low^ population. **(D)** Flow cytometric results of CD8^+^ CD44^Hi^CD62L^Low^, CD8^+^ CD44^Hi^CD62L^Hi^, CD8^+^ CD44^Low^CD62L^Hi^, CD8^+^ CD44^Low^CD62L^Low^, expressed as percentage of tumour-infiltrating CD8^+^ T cells. **(E)** Representative image of CD4^+^CD25^+^CD127^−^ population. **(F)** Flow cytometric results of CD25^+^CD127^-^, expressed as percentage of tumour-infiltrating CD4^+^ T cells. Values are means ± SEM; n = 10 mice/group; *p < 0.05, **p < 0.01, ***p < 0.001 (unpaired t-tests).

As showed in Figure 2, HSF markedly increased the percentage of CD44^Low^ CD62L^Hi^ and CD44^Hi^ CD62L^Hi^ to CD4^+^ T cell populations in the postoperative metastasis mice (Figures 2A,B), and also significantly increased the proportion of CD44^Low^ CD62L^Hi^ and CD44^Hi^ CD62L^Low^ CD8^+^ T cell populations (Figures 2C,D). Additionally, HSF significantly decreased the CD4^+^CD25^+^CD127^-^ population (regulatory T cells) in the tumor environment (Figures 2E,F).

### HSF Inhibited CD8^+^ Tex Cells in the Tumor Microenvironment

**FIGURE 3:**
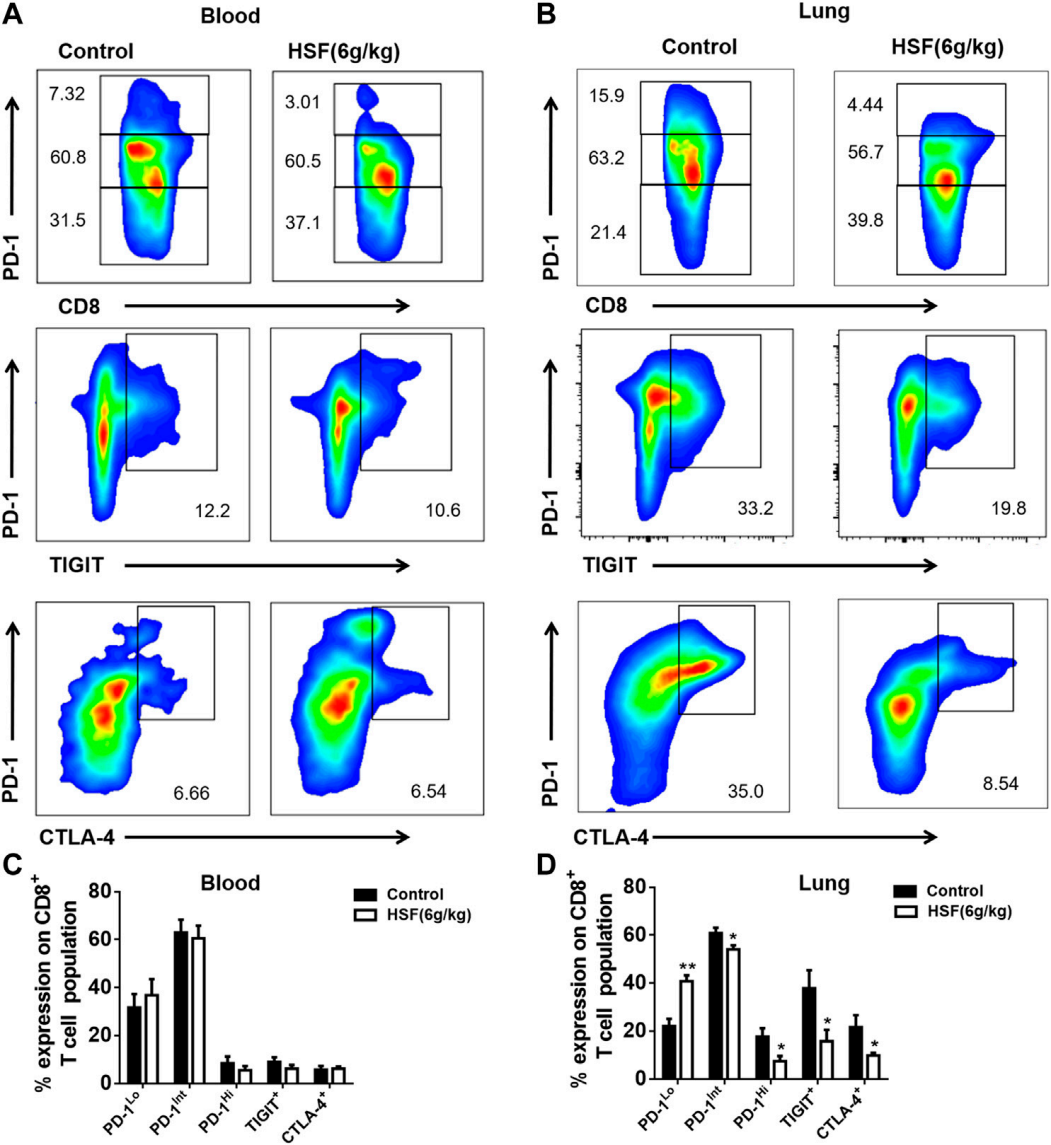
Effect of HSF on PD-1, TIGIT, and CTLA-4 in the blood and lung samples of the breast cancer postoperative metastasis mice. **(A)** Representative image of PD-1, TIGIT, and CTLA-4 population in the blood. **(B)** Flow cytometry of PD-1, TIGIT, and CTLA-4, expressed as percentage of CD8^+^ T cells in the blood. **(C)** Representative image of PD-1, TIGIT, and CTLA-4 population in the lungs. **(D)** Flow cytometry of PD-1, TIGIT, and CTLA-4, expressed as percentage of tumour-infiltrating CD8^+^ T cells in the lungs. Values are means ± SEM; n = 10 mice/group; *p < 0.05, **p < 0.01, ***p < 0.001 (unpaired t-tests).

As shown in Figure 3, the inhibitory receptors of the CD8^+^ T cells from blood, such as PD-1, TIGIT, and CTLA-4, were not significantly different between the HSF and control groups (Figures 3A,B). However, HSF significantly reduced the expression of inhibitor receptors in the CD8^+^ T cells from the lung tumor microenvironment compared to that of the control (Figures 3C,D), and markedly decreased PD-1^Int^ and PD-1^Hi^ CD8^+^ T cell populations. In addition, the results also showed that the inhibitor receptor expression in CD8^+^ T cells was enhanced from the blood to tumor microenvironment.

### HSF Regulated the Expression of the Transcription Factors T-Bet and Eomes in CD8^+^ T Cells

**FIGURE 4:**
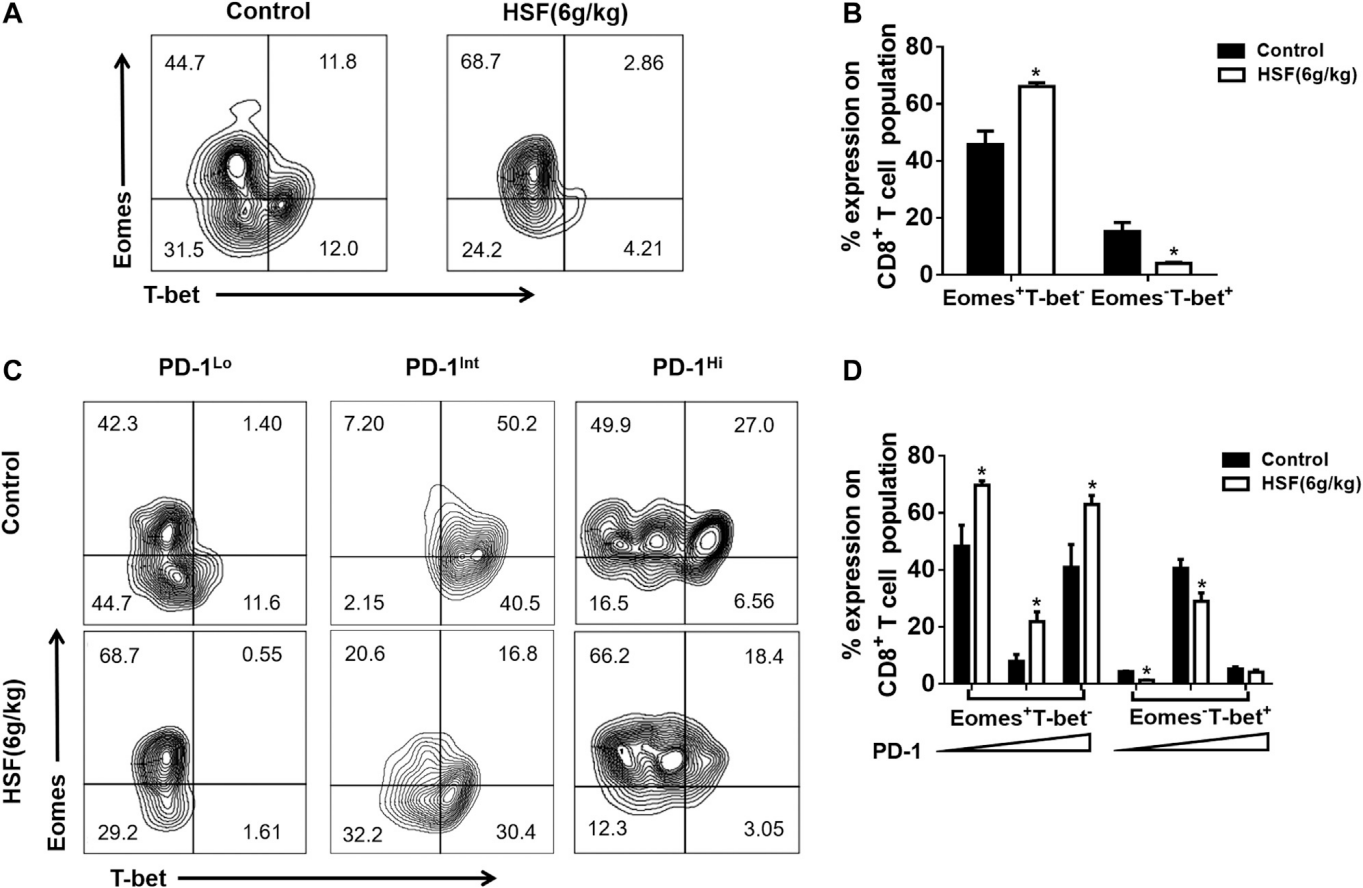
Effect of HSF on Eomes and T-bet expression in exhausted CD8^+^ T cells. **(A)** Representative image of T-bet and Eomes expression on CD8^+^T cells. **(B)** Flow cytometry of T-bet and Eomes, expressed as percentage of lung CD8^+^ T cells. **(C)** Representative image of T-bet and Eomes expression on lung PD-1^Lo^, PD-1^Int^, and PD-1^Hi^ cells. **(D)** Flow cytometry of T-bet and Eomes, expressed as percentage of lung PD-1^Lo^, PD-1^Int^, and PD-1^Hi^ cells. Values are means ± SEM; n = 10 mice/group; *p < 0.05, **p < 0.01, ***p < 0.001 (unpaired t-tests).

T-box transcription factors (T-bet) and Eomesodermin (Eomes) are key to regulating CD8^+^ T cell differentiation and function. We found a very high proportion of Eomes^+^T-bet^-^ expression, while there was a decreased proportion of Eomes^−^T-bet^+^ expression in total CD8^+^ T cells after HSF administration (Figures 4A,B). We further detected Eomes and T-bet expression on PD-1 subpopulations. HSF increased Eomes expression in PD-1^Low^, PD-1^Int^, and PD-1^Hi^ three cell populations, whereas decreased the T-bet expression in PD-1^Low^ and PD-1^Int^ cell populations (Figures 4C,D).

### HSF Inhibited CD8^+^ T Cell Apoptosis and Promoted Proliferation and Function *in vitro*

**FIGURE 5:**
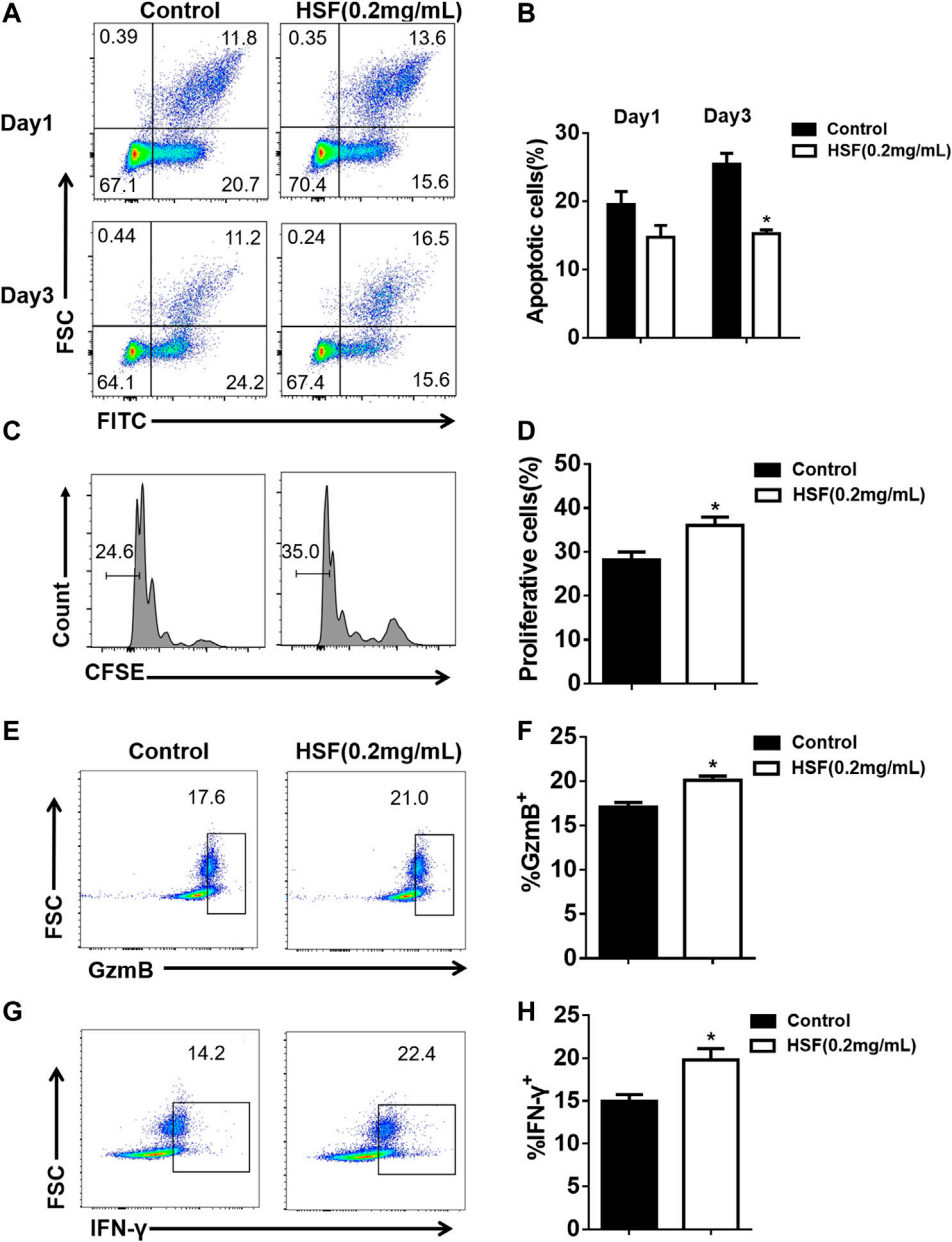
Effect of HSF on the apoptosis, proliferation, and function of CD8^+^ T cells *in vitro*. **(A)** Representative image of apoptotic cells. **(B)** Flow cytometry showing percentage of apoptotic cells among the CD8^+^ T cells. **(C)** Representative image of FACS detecting cell proliferation. **(D)** Statistical analysis of the proliferation percentage of CD8^+^ T cells. **(E)** FACS detection of GzmB production by CD8^+^T cells after 3 days of HSF treatment. **(F)** Flow cytometry of GzmB, expressed as percentage in CD8^+^ T cells. **(G)** FACS detection of IFN-γ production by CD8^+^ T cells after 3 days of HSF treatment. **(H)** Flow cytometry of IFN-γ, expressed as percentage in the CD8^+^ T cells. Values are means ± SEM; *p < 0.05, **p < 0.01, ***p < 0.001 (unpaired t-tests).

The first day after treatment, apoptotic CD8^+^ T cell rate showed no obvious difference between the control (19.5%) and HSF groups (14.7%). However, after 3 days, the HSF group had a significantly lower rate of apoptotic cells (15.2%) than that of the control group (25.5%) (Figures 5A,B). We also found that HSF significantly promoted cell proliferation (Figures 5C,D), and secretion of IFN-γ (Figures 5E,F) and granzyme B (Figures 5G,H) compared to the control.

### HSF Inhibited the Transcription Factor T-Bet in CD8^+^ T Cells *in vitro* and *in vivo*

**FIGURE 6:**
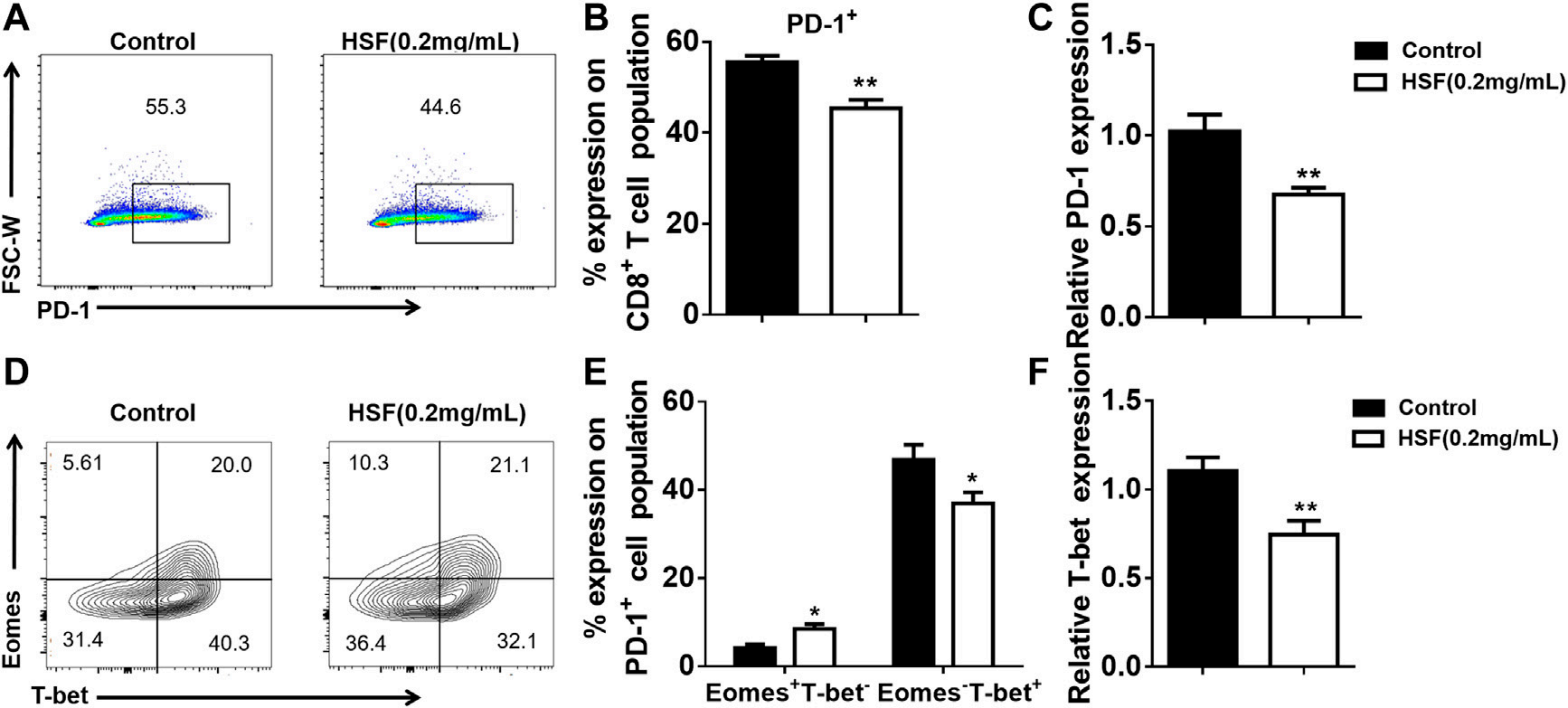
Effect of HSF on *PD-1*, *T-bet*, and *Eomes* expression in CD8^+^ T cells *in vitro*. **(A)** Representative image of PD-1^+^ expression. **(B)** Flow cytometry of PD-1^+^ population, expressed as percentage on the surface of CD8^+^ T cells. **(C)** qRT-PCR of PD-1 expression on CD8^+^ T cells. **(D)** Representative image of T-bet and Eomes expression in CD8^+^ T cells. **(E)** Flow cytometry of Eomes^+^T-bet^-^ and Eomes^−^T-bet^+^ populations as percentage in CD8^+^ T cells. **(F)** qRT-PCR of T-bet expression in HSF-treated CD8^+^ T cells. Values are means ±SEM; *p < 0.05, **p < 0.01, ***p < 0.001 (unpaired t-tests).

HSF decreased the PD-1^+^ levels of CD8^+^ T cells activated with CD3 for 3 days (Figures 6A,B), this result was also confirmed by the mRNA expression assay using qPCR (Figure 6C). Additionally, HSF treatment significantly increased the Eomes^+^T-bet^-^ cell population in CD8^+^ T cells, while it decreased Eomes^−^T-bet^+^ cell population and *T-bet* mRNA expression (Figures 6D–F).

## Discussion

Preventing T cell exhaustion and enhancing CD8^+^ T cell function has become one of the most urgent issues in immunotherapy (Mclane et al., 2019). Our study found that HSF inhibited tumor growth in mice with spontaneous PyMT breast cancer and reduced orthotopic lung metastasis with *in situ* resection of 4T1-luc tumors. These results are consistent with our previous study showing that HSF has anti-tumour effects on lung cancer (Fu et al., 2018). Notably, we also found that HSF has no effect on immunodeficient nude mice. Our results suggest that HSF exerts an anti-tumour effect mainly through the immune system.

**FIGURE 7:**
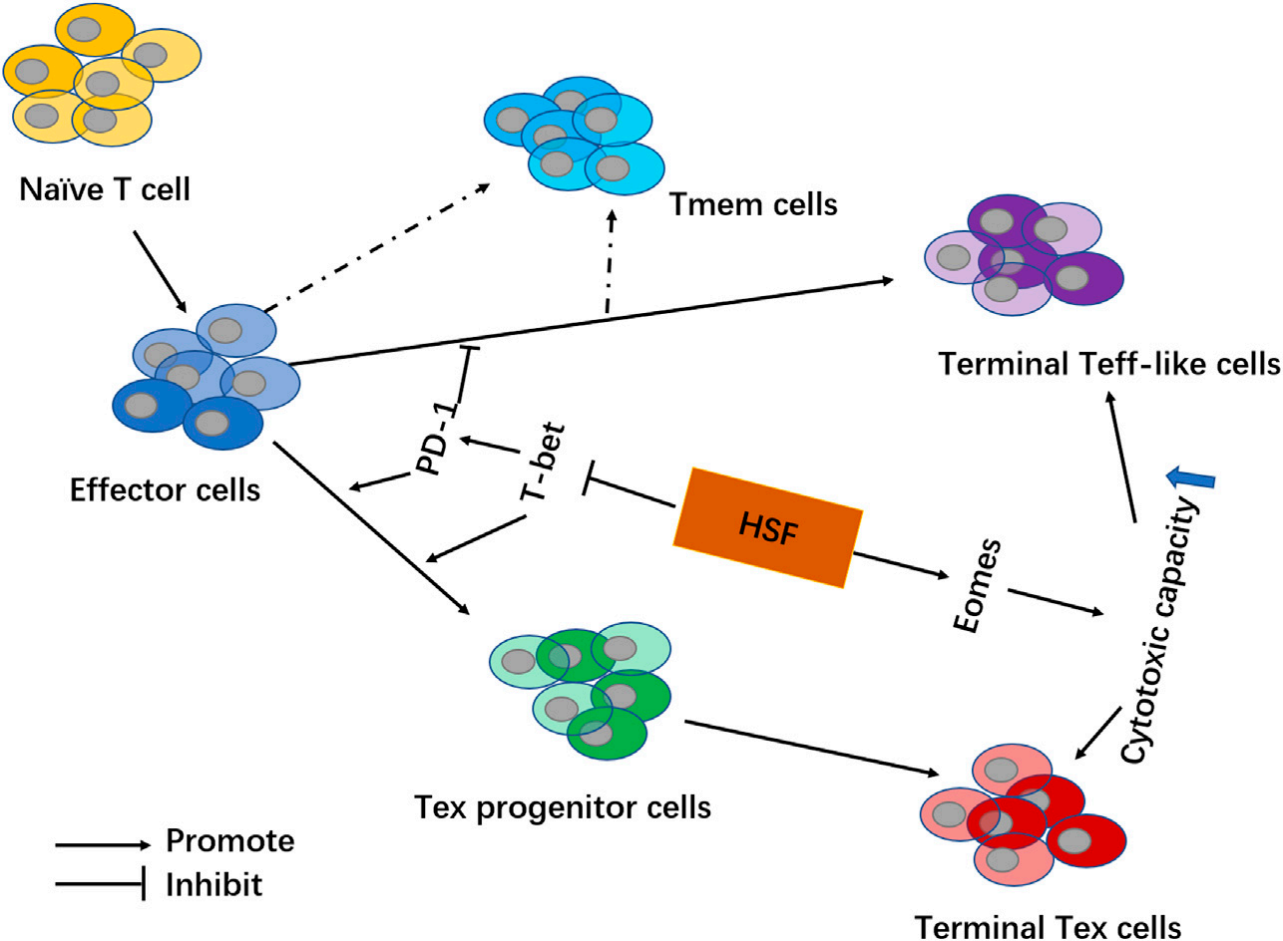
Schematic showing HSF mechanisms involved in regulating exhausted T cells in the tumor microenvironment. By inhibiting transcription factor T-bet, HSF reduces PD-1 expression on T cells and decreases Tex cell formation (including Tex progenitor cells and Terminal Tex cells). Additionally, HSF promotes transcription factor Eomes to increase the secretion of IFN-γ and granzyme B, thereby enhancing the cytotoxic capacity among effector T cells (including Terminal Teff-like cells and Terminal Tex cells).

Accordingly, we further analyzed the T cell subgroups in the tumor microenvironment. CD4 and CD8 memory T cells can be divided into different subgroups according to homing characteristics and effector functions. Central Memory cells (CD44^Hi^CD62L^Hi^, T_CM_) effectively promote the homing of memory T cells to lymphoid organs and respond to antigen secondary stimulation by proliferating and rapidly differentiating into effector T cells. In contrast, effector memory cells (CD44^Hi^ CD62L^Low^, T_EM_) lack lymphoid homing receptors and migrate to non-lymphoid tissues to mediate the ultimate effector function, also called Terminal Teff-like cells (Figure 7). In addition to T_CM_ and T_EM_, recent studies have shown the presence of another type of memory cell in both humans and mice: T memory stem cells (T_SCM_) (Farber et al., 2014; Boudousquié et al., 2014; Chiu et al., 2020). The phenotype of T_SCM_ is CD44^Low^ CD62L^Hi^ SCA-1^Hi^ CD122^Hi^, with strong self-renewal abilities and potential to transform into T_CM_, T_EM_ and effector T cells (Boudousquié et al., 2014; Chiu et al., 2020). In our study, it was found that HSF significantly increased the percentages of effector (CD44^Low^CD62L^Hi^) CD4^+^ T_SCM_ cells, and central memory (CD44^Hi^CD62L^Hi^) CD4^+^ T_CM_ cells, as well as the percentages of (CD44^Low^CD62L^Hi^) CD8^+^ T_SCM_ cells and central memory (CD44^Hi^ CD62L^Low^) CD8^+^ T_EM_ cells, while inhibiting the portion of Tex cells. These effects may be the key mechanism underlying HSF’s ability to significantly improve patient immunity after chemotherapy or surgery.

Tex cells gradually lose effector function and memory T cell characteristics due to prolonged exposure to persistent antigens and inflammation. PD-1 overexpression on CD8^+^ T cells is an important sign of T exhaustion (Pauken and Wherry, 2015a; Wherry and Kurachi, 2015). Tex cells lose their effector function in a progressive and hierarchical manner, depending on antigen levels and disease environment. There is evidence that intermediate PD-1 (PD-1 ^Int^) is expressed in early Tex cells and further differentiated into terminal Tex cells (PD-1 ^Hi^) with a high expression of PD-1 under continuous antigen stimulation, and it is believed that only the PD-1 ^Int^ Tex cells can respond to immune checkpoint inhibitors. Our results showed HSF significantly decreased inhibitory receptor molecules, such as PD-1, Tigit, and CTLA-4, and inhibited these two percentages of PD-1 ^Int^ and PD-1 ^Hi^ CD8^+^ T cell populations. Additionally, our *in vitro* experiments showed HSF promoted T cell expansion, increased IFN-γ and granzyme B secretion, reduced apoptosis, and inhibited PD-1 expression in activated T cells. These results indicate that HSF directly inhibits T cell exhaustion.

Eomes and T-bet are transcription factors that regulate T cell differentiation (Pauken and Wherry, 2015a; Sen et al., 2016). Eomes upregulation is necessary for the formation of long-term memory-like cytotoxic T cells (Pearce et al., 2003). Eomes loss causes defects in memory T cell populations (Chen et al., 2019). In contrast, T-bet induces the production of inhibitory receptor molecules, such as PD-1 and Tigit (Doering et al., 2012). Some evidence has revealed that the Tex cells contain two subpopulations: Tex progenitor cells and terminal Tex cells. Tex progenitor cells highly express T-bet (T-bet^Hi^ PD-1^Int^), which regenerates and maintains virus-specificity through splitting. However, they could differentiate into mature terminal Tex cells expressing Eomes (Eomes^Hi^PD-1^Hi^). This subgroup is more effective against viruses, but cannot self-replicate (Pauken and Wherry, 2015b; Paley et al., 2012; Beltra et al., 2020). Consistent with available literature, we showed that PD-1^Int^ CD8^+^ T cell populations highly expressed T-bet both *in vivo* and *in vitro*. Treatment with HSF then inhibited T-bet expression in CD8^+^ cells, as well as PD-1^Lo^ and PD-1^Int^ cell populations. Thus, HSF regulation of PD-1 is related to T-bet. However, we also found that Eomes was highly expressed in PD-1^Lo^ and PD-1^Hi^ cell populations. Moreover, HSF promoted Eomes expression in CD8^+^ T cells, PD-1^Lo^, PD-1^Int^, and PD-1^Hi^ cell populations. These findings indicate that HSF increases T cell function mainly related to promoting the expression of Eomes (Figure 7).

## Conclusion

Our study demonstrated that HSF exerted anti-tumour effects mainly through effects on the immune system. Specifically, HSF reduced the expression of immune checkpoints through inhibiting T-bet in T cells. This then lowered Tex cell production in the tumor microenvironment. Additionally, HSF promoted Eomes expression to enhance T cell function. These finding provided a new insight into the effects of HSF on tumor immune responses, we recommend that future research investigate the ability of HSF to synergize its effects on immune checkpoint inhibitors.

## Data Availability Statement

The original contributions presented in the study are included in the article/Supplementary Material, further inquiries can be directed to the corresponding authors.

## Ethics Statement

The animal study was reviewed and approved by Ethics Committee of Zhejiang Chinese Medical University.

## Author Contributions

HF and QS conceived and designed the research. QS, LJ (1st author), LJ (2nd author), XL, RW, XZ performed the experiments. HF and LJ (1st author) analyzed the data. HF and QS provided reagents and materials. HF and LJ (1st author) wrote the manuscript.

## Funding

This project was sponsored in part by National Natural Science Foundation of China (81673645, 81873047, and 81573677), the Natural Science Foundation of Zhejiang province (LQ17H030006), Research Fund of Zhejiang Chinese Medical University (2020ZZ01), and Zhejiang Provincial Program for the Cultivation of High-level Innovative Health talents.

## Conflict of Interest

The authors declare that the research was conducted in the absence of any commercial or financial relationships that could be construed as a potential conflict of interest.
